# Applying a video recording, video-based rating method in OSCEs

**DOI:** 10.1080/10872981.2023.2187949

**Published:** 2023-03-08

**Authors:** Yu Fu, Wenjuan Zhang, Saiyi Zhang, Dong Hua, Di Xu, Hua Huang

**Affiliations:** a Oral and Maxillofacial Surgery Medicine, Affiliated Hospital of Stomatology, Nanjing Medical University, Nanjing, China; b Examination management department, National Medical Examination Center, Beijing, China; c Department of Biomedical Engineering and Information, Nanjing Medical University, Nanjing, Jiangsu, China; d Department of Medical Simulation Center, Nanjing Medical University, Nanjing, Jiangsu, China

**Keywords:** OSCE, video recording, on-site rating, assessment, reliability

## Abstract

**Introduction:**

Objective structured clinical examination (OSCE) results could be affected by low homogeneity of examiners, non-retrospectiveness of test results, and examiner-cohort effect. In China, many students participate in medical qualification examinations, and this issue is particularly significant. This study aimed to develop a video recording, video-based rating method and compare the reliability of video and on-site ratings to enhance the quality assurance of OSCEs.

**Methods:**

The subjects of this study were clinical students one year after graduation participating in the clinical skills portion of the National Medical Licensing Examination. The participants were from four cities in Jiangsu province. Participants were randomly allocated to on-site and video rating groups to evaluate the rating methods consistency. We verified the reliability of recording equipment and evaluability of video recording. Moreover, we compared the consistency and equivalence of the two rating methods and analyzed the impact of video recording on scores.

**Results:**

The reliability of recording equipment and evaluability of video recording were high. Evaluation consistency between experts and examiners was acceptable, and there was no difference in evaluation results (P = 0.61). There was good consistency between video and on-site rating; however, a difference between the two rating methods was detected. The scores of video-based rating group students were lower than those of all students (P < 0.00).

**Conclusions:**

Video-based rating could be reliable and offer advantages over on-site rating. The video recording, video-based rating method could provide greater content validity based on its traceability and the ability to view details. Video recording, video-based rating offers a promising mthod for improving the effectiveness and fairness of OSCEs.

## Introduction

The assessment of clinical competence within medical education primarily occurs through written examinations designed to evaluate medical knowledge and performance assessments, such as the objective structured clinical examination (OSCE) to evaluate clinical skills [1,2]. OSCEs are a form of clinical ability assessment with equal emphasis on knowledge, skills, and attitudes. Multi-station assessment methods, standardized patients, virtual patients, computers, and other methods are used to fairly and objectively evaluate the clinical skills of examinees [3]. OSCE assessment results are an essential indicator to evaluate whether medical students possess the professional knowledge and skills necessary for practice [4].

Studies have demonstrated multiple threats to score reliability due to assessment differences [5], with the most problematic sources attributable to the examiner-cohort effect [1]. Harasym et al. examined the influence of examiners’ leniency or harshness on the assessment of communication skills and found that a large amount of variability came from examiners’ systematic biases [6]. Yeates et al. investigated whether groups of parallel examiners in different locations of the same medical school showed different judgment standards and found there were statistically differences in the scoring of the same student ability between different examiner cohorts [7]. Floreck et al. checked scores in 21 areas in the United States and found differences across test locations and between different examiner groups [8]. As the number of students increased, many institutions began to run multiple, parallel, and synchronized OSCE stations. As each student only encounters a single examiner group, any difference in judgment between the examiner groups that affect the pass and fail decisions may affect the fairness and effectiveness of the test. Yeates et al. presented a new method called the Video-Based Examiner Score Comparison and Adjustment and demonstrated that the average scores of some examiner cohorts were quite different [9].

In the physical examination and essential operation skills test stations in China, examinees perform corresponding operational checks, and examiners perform real-time scoring based on the scoring standards and the students’ actual operations. The operation process of students according to the requirements of the test questions is a continuous series of actions. The examiner carefully observes the students’ performance, judges whether the students’ operations are standardized, and then scores based on the grading rules. If the examiner misses or fails to see clearly due to subjective, carelessness or lack of responsibility, or objective factors, such as long work cycle and high intensity, points may be missed or deducted [10], threatening score validity. As such, students’ scores may not reflect their true skill levels. Moreover, as the examination process cannot be traced back, students’ performance cannot be restored; consequently, the objectivity of the examination results cannot be guaranteed.

Examiner training is considered a means to improve test evaluation reliability; however, there may be inconsistencies in the effectiveness of such training [11,12]. As the examiners participating in the operation assessment are from various locations, despite meeting selection criteria, examiners’ professional levels and comprehensive quality differ [13], creating differences in examiners’ assessment quality [14]. Homogeneity in examinations across locations remains low.

Videos have been used in all aspects of medical education. Since the 1980s, videos have been used to assess communication skills [15] and were proven to be effective and reliable in the United Kingdom in the selective assessment of GP trainees [16]. Studies demonstrated that using recorded OSCEs allows examiners to score operation videos on their own time and at any location, improving efficiency, saving time and financial resources, and reducing the number of examiners [16–18]. Moreover, increasing the number of examiners watching the same operation can improve the overall reliability of the test score [5]. In addition, video recording can be used to give feedback to the examinee [19]. Furthermore, it can improve examination results by reducing pressure on examinee in the absence of the on-site examiner [20].

Although video recording has theoretical advantages, in practical applications, the video recording system must accurately capture the details of the operation to prevent students’ bodies or heads from inadvertently blocking the required vital angles. In addition, there are high requirements for the ability of cameras and equipment to achieve high audio and video quality. Furthermore, whether video rating between raters is as reliable as the on-site rating remains unclear.

This study recorded examinees’ practical skills assessment process using the video recording, video-based scoring method and adopted multi-camera synchronization recording to form a video answer sheet for the examinees’ practical skills evaluation. The examiners use the network scoring terminal (referred to as the software platform) to assess videos on three aspects: consistency of evaluation methods, difference in evaluation results, and impact of the video recording system on student scores. This study posed the following questions: (1) Can video recording equipment function reliably? (2) Are scores different between video and on-site ratings? (3) Does the presence of video cameras in the room impact scores? This study aimed to develop a video recording, video-based rating methodology and offer a promising framework for improving the effectiveness and fairness of OSCEs.

## Materials and methods

### Participants

In 2020, 4,714 students participating in the clinical skills portion of the National Medical Licensing Examination from four cities in the Jiangsu province (Nanjing, Suzhou, Nantong, and Xuzhou) were selected. All procedures performed in this study were approved by the ethics committee of Nanjing Medical University and all participants agreed to participate in the study.

### Examination and evaluation methods

The clinical skills portion of the National Medical Licensing Examination consisted of three assessment components, with the first station evaluating clinical thinking. The test questions were delivered by a computer. The test duration of the first station was 40 minutes. The second station evaluated physical examination skills, with a test during of 15 minutes. The third station contained 24 questions on basic clinical skills, including skin disinfection and draping, suturing, abscess incision and drainage, cardiopulmonary resuscitation, etc. The test time was 10 minutes. Candidates completed one question from the second and third station for examination. The physical examination required cooperation of a simulated patient, and basic clinical skill operation was conducted on a simulator or a mold.

### Video collection

The audio and video recording equipment set consisted of a six-camera surrounding unit, a three-camera independent pickup, and a recording and broadcasting workstation to ensure the critical operations were not obscured. During the exam, the audio and video recording equipment documented the complete process, which was integrated into a format with six channels of video and three channels of audio as H.264 format files (frame height 3240, frame width 3840, frame rate 25 frames per second, three audio tracks). The hash value, examinee, examination group, and examination time were calculated, and files were uploaded to the base server. Video answer files were produced. Backup files were stored on the workstation.

### Examiners and scoring

All examiners were experienced clinicians. To improve the homogeneity level of examiners and the efficiency of scoring, the video-based score adopted a classified scoring method, and each examiner only evaluated one type of test. After intensive training, classified training, and test evaluation, the examiners assessed on-site or via video answer files in Nanjing Medical University. Examiners who assessed via video answer files should send their evaluation within three days.

**Figure 1.**
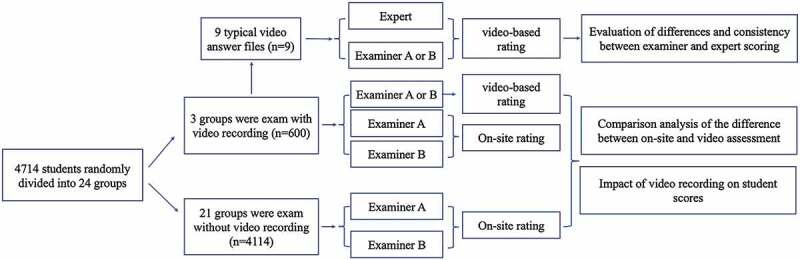
Flowchart of study.

After examination, video answer files were sent to examiners for scoring through the management system network. Each examiner was only assigned one type of exam question. The scoring system provided video viewing angle and audio position switching functions to facilitate examiners to change the audiovisual position. Audio and video could be synchronized fast forward, slow forward, playback, and zoom in any position, and the examiner recorded the scoring results on the structured scoring table. Two examiners independently evaluated each video answer file, and the system automatically submitted the assessment difference to the chief examiner for confirmation to determine whether the evaluation difference exceeded the threshold. The process is represented in a flow chart (Figure 1).

1. Video-based scoring examiner selection and video answer file sampling

As the experimental group, three groups were selected from the second and third stations. The surround recording and broadcasting equipment recorded all students in the experimental group to form a video answer file. Moreover, the examiner was assigned to conduct on-site scoring. In the experimental group, each group selected an examiner to participate in the video-based scoring. The video-based evaluation was performed on 30 randomly selected examination video answer file that the examiner assessed on-site.

2. Sample answer file selection and assessment

Nine typical video answer files were selected from the second and third stations’ video answer files as reference samples. The test experts and examiners chosen from the second and third stations conducted video-scoring of reference samples.

## Reliability of equipment and evaluability of video recording

We counted the number of video answer files that should be received and the number of video answer files actually received in the four bases. Equipment reliability was measured as the number of video answer files actually received divided by the number of video answer files receivable. The examiners evaluated the quality of audio and video recordings of all scoring points during network rating. Evaluability was measured as the number of evaluable scoring points divided by the total number of scoring points.

## Statistical analysis

Measurement data were expressed as the mean ± standard error of the mean (SEM). The non-parametric Wilcoxon rank-sum test evaluated the difference in scores between the two groups with and without video recording in the second and third stations and the difference in video scores between experts and examiners in the second test station. The intra-group correlation coefficient (ICC) was used to evaluate the consistency of the scores of the two video examiners at each station, and the F test was used to test the hypothesis of ICC. ICC is equal to individual variation divided by the total variation with a value belonging to [0,1], where 0 indicates untrustworthy, and 1 indicates completely trustworthy. It is generally believed that a reliability coefficient lower than 0.4 indicates poor reliability, and greater than 0.75 indicates excellent reliability [21]. All tests were performed using Prism 8.00 (GraphPad Software, San Diego, CA, USA) and SPSS version 26 (SPSS, Inc., Chicago, IL, USA). The regular significance level was set to *P* < 0.05, where the adjustment was made according to multi-comparison correction.

## Results

### Reliability and evaluability of video recording

In 2020, the expected number of videos received by candidates from Nanjing, Suzhou, Nantong, and Xuzhou was 1909, 1008, 887, and 910, respectively. The actual number of videos received was 1909, 1008, 887, and 910, respectively, and the missing rate was 0, indicating that the video recording equipment was reliable.

There were 505 scoring points for expert evaluation, 494 scoring points for evaluable points, and the evaluability given by experts was 97.8%. Examiners’ evaluations included 23,107 items, evaluable scores included 22,967 items, and evaluability given by examiners was 99.4%. These results demonstrated that the examiners and experts gave high evaluability to the video record.

### Comparison between examiner and expert scoring

A comparative analysis of the differences between the examiner and expert video scoring results revealed that the average score of examiners was 14.45, and the SD was 2.95. The average score of experts was 14.82, and the SD was 2.60. There was no statistical difference between the results of the examiner and experts for the 18 reference samples (*P* = 0.61; Table 1).

**Table 1.** Score comparison by examiner versus expert assessment (Mean (SD)).

	Score	*P*
Examiner	14.45 (2.95)	0.61
Expert	14.82 (2.60)	

### Consistency evaluation of examiner and expert assessment

Based on 9 sample video observations and scoring, the consistency of the average scores of the 12 examiners and expert scores at the second and third stations was evaluated. The results showed that the average scores of the examiners at the two stations and the expert scores were highly consistent. The ICC was 0.94 (*P* < 0.001) and 0.87 (*P* < 0.001; Table 2). The reliability of the evaluation of the 12 examiners in the second and third stations is illustrated in Table 2. The ICC was 0.83 (*P* = 0.01) and 0.87 (*P* = 0.004), which indicated high reliability in examiners.

**Table 2.** Reliability of scoring of examiner and expert assessment.

	The second test station		The third test station	
	examiners (*n* = 9)	examiners and experts (*n* = 9)	examiners (*n* = 9)	examiners and experts (*n* = 9)
ICC (95%CI)	0.83 (0.23, 0.96)	0.94 (0.84, 0.98)	0.87 (0.43, 0.97)	0.92 (0.80, 0.98)
*P*	= 0.01	<0.001	= 0.004	<0.001

* ICC = intraclass correlation coefﬁcient; 95% CI = 95% conﬁdence interval.

### Comparison analysis of the difference between on-site and video assessment

In clinical skill test, the examiner used a structured score sheet to assess. The results of the second station showed that the average score of video rating was 14.45 (SD = 3.03), which was lower than the on-site rating score (mean = 14.61, SD = 3.06, *P* = 0.046). The results of the third station showed that the video rating score (mean = 13.55, SD = 3.79) was higher than the on-site rating score (mean = 12.49, SD = 4.28, *P* < 0.001; Table 3).

**Table 3.** Scores for on-site and video assessment (Mean (SD)).

	on-site rating	video rating	*P*
The second test station	14.61(3.06)	14.45 (3.03)	0.046
The third test station	12.49 (4.28)	13.55 (3.79)	<0.001

### The impact of video recording on student scores

The difference between the on-site rating results of the students with video recording and the on-site scoring results of all students was analyzed. The results showed that there were differences in the scores between the second station and the third station. The scores of all students group were 15.09 (SD = 3.37) and 13.41 (SD = 3.99) on the second and third test station, respectively, which were both higher than the scores of video recording group (mean = 14.61, SD = 2.99, mean = 12.49, SD = 4.24; *P* < 0.00; Table 4).

**Table 4.** Scores for the video test group and the general test group assessment (Mean (SD)).

	all students		Video recording		*P*
	N	Score	N	Score	
The second test station	9196	15.09 (3.37)	461	14.61 (2.99)	<0.00
The third test station	9196	13.41 (3.99)	468	12.49 (4.24)	<0.00

## Discussion

The clinical skill test is an integral part of the physician qualification exam in China. At present, the clinical skill test mainly takes the form of on-site examiner scoring. However, the traditional examiner grading model has certain shortcomings. The test process cannot be reproduced, and test scores cannot be traced back and verified. Some examiners are not strictly controlled, which could lead to undesirable validity and consistency. The organization of assessments requires a large number of experienced physicians to serve as examiners, which brings considerable pressure to the clinical work of local hospitals. Clinical skills test uses a multi-channel OSCE test station, which makes it difficult to achieve homogeneity of evaluation. Examiners in the same test group have to evaluate multiple examination questions, which causes the examiner to be more stressed.

Video rating is a digital examination evaluation mode that records the student’s performance process in a clinical skill test. This progress is then played back through network and multimedia equipment, and the examiner performs comprehensive scoring according to structured scoring standards. The aim of video rating is to subjectively eliminate the examination view of emphasizing results and neglecting process, establish an examination view that emphasizes both results and process, and appropriately highlight process. During our study, we found that video-based rating offers potential advantages in OSCE. One of these advantages is that the recorded video can be used as effective learning resource for students to intuitively understand the problems that occur during OSCE. Another advantage of video rating is quality control. Due to traceability and detailed inspection, examiners can assess exam skills according to standardized grading criteria, which can improve the objectivity of assessments.

One of the most important factors of OSCEs scoring is reliability. Vivekananda-Schmidt et al. investigated the inter-rater reliability of OSCE stations for shoulder and knee examinations of 95 third-year medical students between real-time and video-recorded evaluations and found that the scores may not be equivalent [21]. Sturpe et al. examined internal reliability issues between real-time observation and video-based observation and found that 13.3% of students who passed based on real-time observation were evaluated as failed based on video observation by the same rater, whereas 3.3% of students who failed in real-time observation passed under video observation [17]. In this study, the scoring of the two evaluation methods was compared and analyzed to determine whether the video evaluation method can replace the on-site rating method in clinical skill tests. As video records can be reviewed repeatedly, and score points cannot be missed, this may have caused lower video rating results than on-site rating results in the second station. This suggests that the video recording and video-based rating method could be convenient for clearly identifying students’ operational errors and producing an objective evaluation. In this study, the examiner for the third station did not fully understand the aseptic scoring criteria, resulting in repeated deductions, which may have caused the on-site rating score to be lower than video scoring. These results suggest that the application of video-based rating method could establish retrospective and verifiable examination records for students and reproduce the examination process, providing a basis for the reliability of examination evaluation.

When designing video recording, vital issues, such as necessary equipment and professional knowledge, need to be considered to obtain high-quality audio and video data. Nanjing Medical University has continuously improved the audio and video collection programs and technologies. This study used a 360-degree surround video camera supplemented by three independent pickups to collect stable and clear pictures, high-quality sounds, and wide angles to meet the needs of examination evaluation. In addition, video recording could be an essential reference material for students to improve their operational skills. However, the performance of students may be affected by the video recording process [21]. This study did not delve into students’ attitudes toward video recording. Through a comparative analysis of the on-site rating scores of students who set up video equipment at the second and third station and on-site rating scores of all students, the impact of the video recording on the test scores was evaluated. Lower on-site rating results of students with video recording than those of all students may be due to students being nervous when facing the video recording, which slightly impacted the test scores. These results imply that the examinees’ familiarity with the video recording system should be strengthened in the application process. Corresponding explanations and adaptive preparations should be done before the test.

Furthermore, this study identified additional advantages of video rating. Examiner evaluations and student examinations did not need to be performed simultaneously, increasing flexibility in scheduling and reducing time constraints. The efficiency of video-based rating was also higher than that of on-site rating, alleviating pressure on examiners. Each examiner was assigned only one type of examination question via video-based rating method, significantly reducing the pressure of scoring and improving assessment efficiency. Additionally, each examination question was independently evaluated by two examiners, and the system automatically submitted any differences exceeding the threshold to the chief examiner for confirmation evaluation, enhancing evaluation homogeneity.

## Limitations

Although our study showed that video recording and video-based rating had significant strengths in OSCEs, they nonetheless have some limitations. We conducted a statistical analysis to determine whether video recording had an impact on the scores. Although we did not find a significant effect on the scores, further research is needed to improve this conclusion. This could include questionnaire analysis of participants to determine whether the video recording affected their performance. Additionally, the examiner’s feedback on the experience of using the video evaluation, including operability, video clarity, and evaluability, are also important indicators for evaluating the application of the video recording and video-based rating method.

## Future study

Future studies should address the limitations described. The video recording process needs further optimization to ensure the clarity and evaluability of the recording details, providing high-quality video information to the examiner. Studies should explore the perceptions of examiners and participants on the use of video recording, video-based rating method, and the impact of interventions on assessment behavior. Further research should be conducted on the impact of video recording feedback on teaching levels and candidates’ ability improvement. Additionally, more studies on the objectivity and evaluability of video-based rating methods need to be explored and refined.

## Conclusions

The traditional scoring model is not sufficiently objective and efficient. This study revealed that the video rating mode could eliminate the examiner’s intervention with the students on-site and improve the objectivity and fairness of examinations. Moreover, the method of categorized scoring could reduce examiners’ work pressure, improve examiners’ efficiency, and evaluation homogeneity, ensuring an objective and effective evaluation of students’ clinical skills. Therefore, it is necessary to extend this model to practicing physician qualification examinations.

## Data Availability

The data that support the findings of this study are available from the corresponding author, Hua Huang, upon reasonable request.
